# Plague Exposure in Mammalian Wildlife Across the Western United States

**DOI:** 10.1089/vbz.2020.2765

**Published:** 2021-09-16

**Authors:** Sarah N. Bevins, Jeffrey C. Chandler, Nicole Barrett, Brandon S. Schmit, Gerald W. Wiscomb, Susan A. Shriner

**Affiliations:** ^1^ USDA APHIS WS National Wildlife Research Center, Fort Collins, Colorado, USA.; ^2^ USDA APHIS WS National Wildlife Disease Program, Fort Collins, Colorado, USA.; ^3^ USDA APHIS Wildlife Services Montana, Billings, Montana, USA.

**Keywords:** *Yersinia pestis*, spillover, pathogen, sentinel, coyote

## Abstract

Plague is caused by a bacterial pathogen (*Yersinia pestis*) that can infect a wide range of mammal species, but its presence in wildlife is often underappreciated. Using a large-scale data set (*n* = 44,857) that details the extent of *Y. pestis* exposure in wildlife, we document exposure in 18 wildlife species, including coyotes (*Canis latrans*), bobcats (*Lynx rufus*), and black bears (*Ursus americanus*). Evidence of plague activity is widespread, with seropositive animals detected in every western state in the contiguous United States. Pathogen monitoring systems in wildlife that are both large scale and long-term are rare, yet they open the door for analyses on potential shifts in distribution that have occurred over time because of climate or land use changes. The data generated by these long-term monitoring programs, combined with recent advances in our understanding of pathogen ecology, offer a clearer picture of zoonotic pathogens and the risks they pose.

## Introduction

Plague is a disease caused by the bacterial pathogen *Yersinia pestis*, and although it is often closely associated with historic human pandemics, *Y. pestis* is primarily a pathogen of rodents and other wild animals. Its presence in wildlife populations often goes unrecognized but can be extensive.

Plague is largely maintained through *Y. pestis* transmission between rodent hosts and their fleas (Macchiavello 1954, Pollitzer 1954), with some rodent species developing asymptomatic infections, whereas others suffer severe morbidity and mortality. Spillover into other taxa can also result in clinical disease and death, with humans, felids, lagomorphs, and prairie dogs being some of the species known to develop severe *Y. pestis* infections (Eidson et al. 1988, Gasper et al. 1993, Gage et al. 1994, Biggins and Kosoy 2001, Gage and Kosoy 2005). Clinical disease in susceptible hosts manifests as bubonic, septicemic, or pneumonic plague, and infections that are not treated with antibiotics remain as deadly as they were hundreds of years ago (Craven et al. 1993).

Human plague outbreaks still occur in multiple regions around the world (Vallès et al. 2020), but in the United States, the human health burden is often low, with an average of seven cases a year (Kwit et al. 2015). Although the public health burden is low, the wildlife health burden can be substantial. There are significant wildlife and domestic animal health issues associated with plague, the best known of which are the dramatic die-offs seen in prairie dogs (*Cynomys* sp.) and black-footed ferrets (*Mustela nigripes*), where mortality can approach 100% for both predator and prey (Matchett et al. 2010, Biggins and Eads 2019). Black-footed ferrets are an endangered species whose reintroduction efforts are continually complicated by plague outbreaks in the western United States. Plague-associated mortality is also seen in wildlife species such as mountain lions (*Puma concolor*; Bevins et al. 2009) and lynx (*Lynx canadensis*; Wild et al. 2006), but other predators, such as coyotes (*Canis latrans*) and foxes (*Vulpes* spp.), readily survive exposure, often showing little to no clinical signs of infection (Baeten et al. 2013). Exposure to plague in these predator species likely occurs after they prey on rodents that harbor *Y. pestis*, or through the bites of *Y. pestis*-positive fleas acquired from rodent hosts. Domestic cats and dogs can also be infected and are routinely a source of human infections in the United States (Gage et al. 2000, Wang et al. 2011).

Considering the human, domestic animal, and wildlife health risks associated with plague, there is a need to better understand plague distribution on the landscape, especially in wildlife. Robust data are available on human infections in the United States (Eisen et al. 2007, Kwit et al. 2015) and there are rich wildlife data associated with visible prairie dog mortality events (Matchett et al. 2010, Biggins and Eads 2019), but broadscale information available on plague distribution in wildlife is more limited (Barnes 1982). A state level meta-analysis on plague in wild carnivores found substantial variation in exposure (Salkeld and Stapp 2006) and wildlife plague data have been used to highlight the likely role of flea vectors in determining the geographic distribution of plague in the United States (Maher et al. 2010), but gaps remain despite over a century of plague research in the United States.

In this study, we expand on that work using a long-term, large-scale data set (*n* = 44,857 samples) collected from wildlife across the western United States to better understand *Y. pestis* distribution and exposure. These data primarily focus on documenting exposure in coyotes and other carnivore species because, although they only rarely contribute to *Y. pestis* transmission dynamics—they typically have asymptomatic infections and do not necessarily develop a high enough bacteremia to infect feeding fleas—these species develop long-lived antibody responses after being exposed to *Y. pestis* and therefore can act as sentinels of pathogen activity on the landscape. Using data from wildlife sentinels collected opportunistically, but consistently, and at scale, can provide a clearer picture of plague in the American West.

## Methods

Samples were collected between 2005 and 2018, in cooperation with state and federal agencies, throughout the western United States. Collection was opportunistic, in conjunction with permitted wildlife damage management activities, other wildlife management activities (*e.g.*, re-introductions), or wildlife mortality investigations. Samples from various carnivores, especially canids, were prioritized for collection and testing because of previously published research showing high levels of *Y. pestis* exposure in generalist carnivores (Gage et al. 1994, Brown et al. 2011). For sample collection, Nobuto filter paper strips were saturated with whole blood and then allowed to air dry before being shipped in a sealed plastic bag with desiccant packs. Samples collected after 2012 were stored at −20°C to enable long-term storage before testing (Bevins et al. 2016). Multiple Nobuto strips were collected from each animal, with extra strips archived in the National Nobuto Sample Archive housed at the USDA APHIS WS National Wildlife Disease Program in Fort Collins, CO. Metadata associated with each sample included sample date, species sampled, and latitude/longitude of collection location. County of sample collection was determined by collection location.

Nobuto filter strip samples were screened for antibodies to the *Y. pestis* F1 antigen using two different assays. Most samples collected before 2012 and most California samples were tested using a passive hemagglutination assay (PHA; Chu 2000). These samples were also tested for hemagglutination inhibition to screen out samples demonstrating nonspecific binding (Chu 2000). Samples had to display a titer ≥1:32 to be considered PHA positive (Chu 2000). Most canid samples collected after 2012 were screened for *Y. pestis* using a recently developed bead-based flow cytometric assay (F1 Luminex plague assay), which has been validated for canids. This assay is based upon Luminex/Bio-Plex technology and, like the PHA, targets the serological response to the F1 capsular antigen of *Y. pestis* (Chandler et al. 2018). All noncanid species were tested using the PHA assay only. The strengths and weaknesses of each assay have been previously characterized (Marshall et al. 1972, Chandler et al. 2018). Individual samples were only tested using one assay or the other, not both.

## Seroprevalence Estimation

True seroprevalence that accounts for the sensitivity and specificity of each assay was estimated for each species and for each county using a Bayesian approach. Prior information on sensitivity and specificity of the two assays was determined using published data on experimental infections (Marshall et al. 1972, Baeten et al. 2013, Chandler et al. 2018) and based on expert opinion (Table 1). Published estimates of sensitivity and specificity were transformed to beta parameters (α, β) using BetaBuster (Su 2006). True mean prevalence was calculated using package “prevalence” version 0.4.0 built for R version 3.5.3 (Devleesschauwer et al. 2014, R Core Team 2019) for each species group and for canids in each county. Prevalence, sensitivity, and specificity can vary between 0 and 1, so informed priors used a beta distribution to model the uncertainty within this space. We used the default settings of 100,000 iterations after a discarded burn-in of 10,000 iterations. Convergence was assessed using the Gelman–Rubin–Brooks statistic. For counties that had samples tested using both assays, true prevalence was weighted by the number of samples for each assay in that county, and then averaged for the two assays to produce a single estimate of true prevalence. True prevalence calculations were completed on counties and species with ≥1 positive detection; counties or species with no positive detections were assumed to have a prevalence of 0%.

**Table 1.** Published Estimates Used to Determine Informative Priors

	Passive hemagglutination assay		Bead-based flow cytometric assay	
	Sensitivity	Specificity	Sensitivity	Specificity
Estimate	0.81	0.98	0.94	0.96
Citation	Marshall et al. (1972)	Marshall et al. (1972)	Chandler et al. (2018)	Chandler et al. (2018)
Prior	Beta (95% sure that se >0.6 with mode = 0.81)	Beta (95% sure that sp >0.8 with mode = 0.98)	Beta (95% sure that se >0.85 with mode = 0.94)	Beta (95% sure that sp >0.7 with mode = 0.96)
A	13.677	15.798	23.788	10.199
B	3.954	1.302	2.454	1.383

## Findings

From 2015 to 2018, 44,857 samples were collected from wildlife in the western United States and screened for exposure to *Y. pestis*. Much of this surveillance focused on canids and other predators in the order Carnivora and they, especially coyotes, comprised the majority of samples. Specifically, 92.81% (*n* = 41,632) of samples were from species in the order Carnivora, and 84.47% (*n* = 37,892) were from canids. Coyotes accounted for 79.95% (*n* = 35,866) of samples (Table 2).

**Table 2.** Samples Sizes and True Seroprevalence of Wildlife Species Screened for *Yersinia pestis* Antibodies

Species	Scientific name	n	% Mean true prevalence (95% credible interval)
Wolverine	*Gulo gulo*	1	63.9 (10–99)
Kit Fox	*Vulpes macrotis*	12	18.8 (1–54)
Gray wolf	*Canis lupus*	551	14.3 (3–23)
Black-tailed Prairie dog	*Cynomys ludovicianus*	32	11.2 (1–31)
Gray fox	*Urocyon cinereoargenteus*	248	9.8 (1–21)
Coyote	*Canis latrans*	35866	8.5 (1–17)
American Badger	*Taxidea taxus*	159	7.0 (0–16)
Swift fox	*Vulpes velox*	23	6.7 (0–21)
Black bear	*Ursus americanus*	486	4.3 (0–10)
Bobcat	*Lynx rufus*	366	3.2 (0–8)
Mountain lion	*Puma concolor*	388	3.2 (0–8)
Red fox	*Vulpes vulpes*	531	3.1 (0–8)
Feral swine	*Sus scrofa*	484	2.0 (0–5)
Cottontail rabbit	*Sylvilagus*	183	1.0 (0–4)
Other		853	0.5 (0–2)
Striped skunk	*Mephitis mephitis*	698	0.5 (0–2)
Raccoon	*Procyon lotor*	1386	0.4 (0–1)
North American beaver	*Castor Canadensis*	1568	0.1 (0–1)

Species with 0 positives: Nutria (*Myocastor coypus*, *n* = 200), jackrabbit (*Lepus* sp. *n* = 186), muskrat (*Ondatra zibethicus*, *n* = 88), desert woodrat (*Neotoma lepida*, *n* = 65), Virginia opossum (*Didelphis virginiana*, *n* = 64), hooded skunk (*Mephitis macrouran*, *n* = 39), deer mouse (*Peromyscus maniculatus*, *n* = 34), cactus mouse *(Peromyscus eremicus*, *n* = 30), North American river otter (*Lontra canadensis*, *n* = 25), rock squirrel (*Otospermophilus variegatus*, *n* = 24), Merriam's kangaroo rat (*Dipodomys merriami*, *n* = 23), Pinyon mouse (*Peromyscus truei*, *n* = 23), round-tailed ground squirrel (*Xerospermophilus tereticaudus*, *n* = 20), black rat (*Rattus rattus*, *n* = 19), Fisher (*Pekania pennanti*, *n* = 18), eastern cottontail rabbit (*Sylvilagus floridanus*, *n* = 15), California ground squirrel (*Otospermophilus beecheyi*, *n* = 14), white-throated woodrat (*Neotoma albigula*, *n* = 14), house mouse (*Mus musculus*, *n* = 13), white-lipped peccary (*Tayassu pecari*, *n* = 13), kangaroo mouse (*Microdipodops*, *n* = 11), yellow-bellied marmot (*Marmota flaviventris*, *n* = 11), brown rat (*Rattus norvegicus*, *n* = 10), ground squirrel (*Marmotini* sp. *n* = 9), western spotted skunk (*Spilogale gracilis*, *n* = 8), American pygmy shrew (*Sorex hoyi*, *n* = 6), North American porcupine (*Erethizon dorsatum*, *n* = 6), grizzly bear (*Ursus arctos horribilis*, *n* = 3), white-tailed antelope squirrel (*Ammospermophilus leucurus*, *n* = 3), black-tailed jackrabbit (*Lepus californicus*, *n* = 2), brush mouse (*Peromyscus boylii*, *n* = 2), bushy-tailed woodrat (*Neotoma cinerea*, *n* = 2), collared peccary (*Pecari tajacu*, *n* = 2), Douglas squirrel (*Tamiasciurus douglasii*, *n* = 2), mule deer (*Odocoileus hemionus*, *n* = 2), Panamint chipmunk (*Tamias panamintinus*, *n* = 2), Townsend's mole (*Scapanus townsendii*, *n* = 2), western harvest mouse (*Reithrodontomys megalotis*, *n* = 2), western hog-nosed skunk (*Conepatus leuconotus*, *n* = 2), antelope squirrel (*Ammospermophilus*, *n* = 1), fox squirrel (*Sciurus niger*, *n* = 1), long-tailed weasel (*Mustela frenata*, *n* = 1), Mexican wolf (*Canis lupus baileyi*, *n* = 1), Pacific jumping mouse (*Zapus trinotatus*, *n* = 1), pronghorn antelope (*Antilocapra americana*, *n* = 1), stoat (*Mustela erminea*, *n* = 1), white-tailed deer (*Odocoileus virginianus*, *n* = 1).

**FIG. 1.**
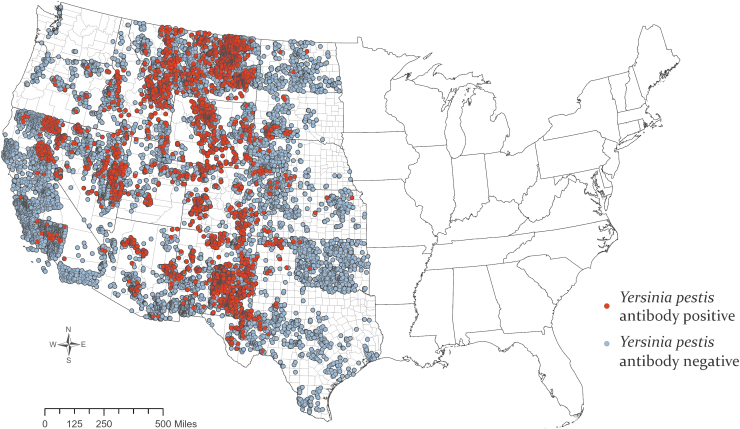
Plague seropositive and seronegative samples collected from wild canids, 2005–2018. Color images are available online.

**FIG. 2.**
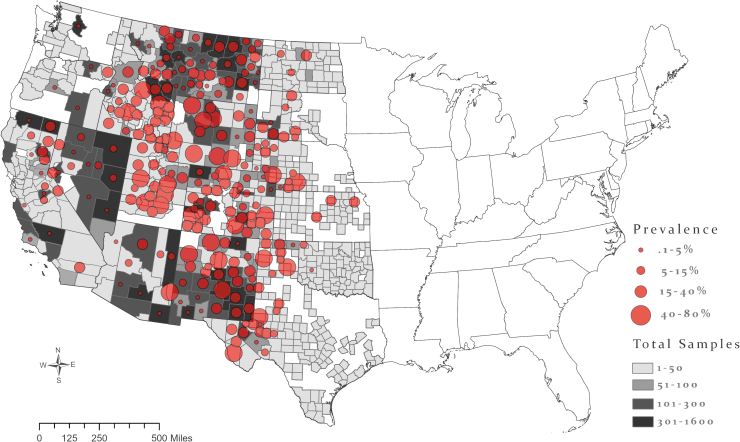
True seroprevalence of canid samples collected from 2005 to 2019. *Gray* county shading shows the number of samples tested in that county. Diameter of *circles* shows county seroprevalence. Color images are available online.

Accurate county-level location data were available for 37,892 canid samples, and mapping revealed widespread *Y. pestis* exposure (Fig. 1). Overall, 7% (95% Credible Interval [CrI], 0.38–14.9) of sampled individuals were seropositive (Fig. 2). Wildlife were exposed to *Y. pestis* in every state in the western half of the contiguous United States. New Mexico and the Four Corners region (Arizona, Colorado, New Mexico, and Utah) are where a majority of human cases occur in the United States (Kwit et al. 2015) and a similar signal was seen in this study, which also demonstrated substantial *Y. pestis* exposure in wildlife in New Mexico (20.5%; 95% CrI, 6.4–65.7); however, other states that are only rarely associated with human plague cases also had high seroprevalence in wild canids, including Wyoming (28.1%; 95% CrI, 10.7–49.1) and Utah (20.6%; 95% CrI, 3.5–41.9).

At the county level, the three counties with the highest *Y. pestis* seroprevalence in wild canids and that had 30 or more samples collected include San Miguel County, CO (79.5%; 95% CrI, 54.5–98.1; *n* = 45), Carbon County, WY (73.9%; 95% CrI, 45.5–97.2; *n* = 60), and Park County, WY (67.4%; 95% CrI, 44.4–94.2; *n* = 79), although 120 counties in this dataset had a true seroprevalence of 20% or more (Fig. 2). Lincoln County, NM was one of the most well-sampled areas in this data set, resulting in robust sample coverage throughout the period of the study (*n* = 1569), and a seroprevalence of 46.3% (95% CrI, 22.9–66.6) in canids (Fig. 2).

Across the western United States, mean true prevalence for coyotes, the most frequently sampled species, was 8.6% (95% CrI, 0.5–17.4). Other frequently sampled carnivore species exposed to *Y. pestis* (Table 2) include the gray wolf (*Canis lupus*: 14.3%, 95% CrI 3.5–22.8) and gray fox (*Urocyon cinereoargenteus*: 9.8%; 95% CrI, 0.7–21.1). A number of other species also had high seroprevalence values, but in most cases, sample sizes were low for these species (Table 2), which precluded precise seroprevalence estimates. The complete list of counties and species sampled provides a full accounting of the data, but many of these had only a limited number of samples available for testing, so definitive conclusions on the frequency of exposure cannot be drawn (Table 2).

## Discussion

This comprehensive data set documents the extent of *Y. pestis* exposure in wildlife species found across the western United States and the resulting information is available for use by wildlife managers, public health departments, and others to better understand plague occurrence in their area. Analyzing these data as a whole demonstrates widespread evidence of plague, reinforcing the idea that although it is an introduced pathogen in the United States (Eskey and Haas 1939), it is a persistent presence in wildlife. The overall seroprevalence in wildlife was 7%, but some counties had substantially higher exposure, with >70% of animals showing evidence of *Y. pestis* antibodies.

The idea of continual *Y. pestis* transmission across the landscape is not new (Barnes 1982, Biggins and Kosoy 2001, Biggins et al. 2010); however, the extent of exposure in wildlife is underappreciated because few data sets have been available to examine exposure at scale. The magnitude of animal exposure is reinforced by original research by Barnes (1982) and by studies showing that survival in prairie dog colonies increases when flea control products are applied, even in the absence of visible plague activity (Biggins et al. 2010). In addition, vaccination of black-footed ferrets against plague resulted in a >200% increase in survival, again, when there was little evidence of plague transmission on those colonies (Matchett et al. 2010). The increased survival in this and other studies (Biggins et al. 2021, Goldberg et al. 2021) is likely indicative of persistent, low-level *Y. pestis* transmission that is difficult to detect; transmission that primarily occurs outside the scope of traditional data sets on human case investigations and visible wildlife mortality events.

The degree to which wild animals are exposed to *Y. pestis* contrasts with the limited number of cases in humans. It is clear that most wildlife-to-human transmission in the United States occurs when wild rodents enter human settlements or the surrounding area, and this risk increases following rodent population booms. Rodent population booms often follow pulses in primary productivity, but once resources are depleted, rodent populations collapse (or expand into human settlements), forcing their fleas to find alternative hosts. This also occurs when plague epizootics kill off large number of rodents. Regardless of the proximate mechanism, these chain-of-events can ultimately bridge the divide between wildlife and people. In the absence of these situations, enzootic plague exists in wild animal populations, sometimes at relatively high levels, separate from the spillover risk to humans. Although often not directly correlated, plague in humans and plague in wildlife are still inexorably linked. Samia et al. (2011) noted that understanding the human component of plague is impossible in isolation from the dynamics of the wild animals involved in plague transmission. Although human plague cases are uncommon in the United States, they can still represent a public health burden. A single domestic animal case recently resulted in the potential exposure of >100 people and 46 animals at a veterinary hospital and those exposures required contact tracing and postexposure prophylactic antibiotics (Schaffer et al. 2019).

### Detecting antibodies in sentinel species

These results also reinforce the utility of using predators as sentinel species of plague occurrence at large spatial scales. *Y. pestis* antibodies were detected in 18 species in this data set, showing the extent to which different species can be exposed to the pathogen; however, a majority of samples were from canids and other predators. Although canids can occasionally play a role in *Y. pestis* transmission (Poland et al. 1973, Wang et al. 2011) and they have been implicated in moving infected fleas around the landscape (Maher et al. 2010), they are generally considered refractory to infection (although see Orloski and Eidson 1995, Nichols et al. 2014, Schaffer et al. 2019). Despite their relatively limited contribution to transmission, screening these species as sentinels is beneficial because (i) they do not typically succumb to plague infections and therefore are available to sample, (ii) they develop long-lasting antibodies that leave a record of *Y. pestis* exposure, and (iii) they move over larger spatial scales than the plague-positive rodents that make up part of their prey base, essentially sampling the environment and providing a broad indication of plague activity on the landscape. These larger spatial extents are an efficient way to detect a *Y. pestis* signal when examining general geographic patterns; however, the converse is also true, in that antibody presence in these sentinel species reveals less about local-scale infection risk (Brinkerhoff et al. 2009). Caution must also be exercised when comparing seroprevalence in sentinel species across different regions. Regional differences in plague activity certainly exist, but with sentinel species exposed to *Y. pestis* through prey consumption, differences in seroprevalence could be confounded by a difference in regional prey species as well. Despite these difficulties, monitoring plague exposure in predators has proved valuable. Positive coyote samples have been linked to areas of predicted rodent infection (Holt et al. 2009) and to human plague cases (Brown et al. 2011), suggesting that increasing seropositivity rates in carnivores may be indicative of an increase in spillover risk.

Antibodies are detectable for a longer period of time than are pathogen antigens in the host, making them a good target for wildlife pathogen monitoring programs. The window of time to detect an active infection is often days to weeks (Rust et al. 1971, Zhang et al. 2012), and in the case of coyotes and *Y. pestis*, bacteria may never replicate to high enough levels to be detectable using antigen-based assays (Baeten et al. 2013). The persistent nature of these antibodies, for example, dogs displaying stable *Y. pestis* antibody levels 330 days postexposure (Rust et al. 1971), increases the chances of detecting a disease signal during a single wild animal sampling event. This, combined with the fact that many of these sentinel species are long-lived compared with rodents (coyotes can live >10 years), increases the chances of an individual being exposed or re-exposed during the course of their life and concomitantly, increases the chances to detect evidence of *Y. pestis* on the landscape. The disadvantage with carnivore serosurveys is that antibody presence cannot tell you when an animal was infected, information that can be critical when attempting to pinpoint the ecological mechanisms that drive pathogen transmission (Gilbert et al. 2013, Pepin et al. 2017). The potential for repeated exposure to an enzootic pathogen and a boosted antibody response further limits the ability to determine time of infection. If the goal is mechanistic understanding, some of the difficulties associated with antibody data can be overcome with a combination of long-term experimental infections that provide valuable information on the duration of antibody responses, combined with quantitative assays that characterize the strength of the antibody response. When this information is absent, seroprevalence is a poor metric for quantifying pathogen dynamics.

### Mechanisms associated with spillover

Pinpointing the mechanisms that drive *Y. pestis* transmission and understanding where it resides in between visible outbreaks in wildlife, domestic animals, or humans has been a long sought-after goal of plague research. The hypothesized mechanisms governing the triumvirate of a vector/host/pathogen system are, of course, complex. Rodent host abundance and diversity are thought to be key, with abundance thresholds highlighted as a key driver (Davis et al. 2004) and percolation dynamics potentially governing transmission through metapopulations of rodent hosts (Davis et al. 2008, Salkeld et al. 2010). Flea vectors play a key role in maintaining plague on the landscape and in spillover dynamics to humans. Specific flea-rodent assemblages likely determine enzootic plague distribution around the world (Maher et al. 2010). Climate also plays a role, with time-lagged increases in plague activity seen after precipitation events in semi-arid systems (Enscore et al. 2002, Stenseth et al. 2006, Ben Ari et al. 2011, Schmid et al. 2015). There have also been recent advances in our understanding of *Y. pestis* and its potential persistence in soils (Eisen et al. 2008, Barbieri et al. 2020). The importance of these factors may not be uniform across the regions of the world where plague occurs, making generalities difficult and robust predictions problematic. Each of these variables may be involved in pathogen persistence on the landscape or they may be crucial drivers in the explosive pathogen growth seen during epizootics or epidemics. But scale matters (Ben Ari et al. 2011, Eads and Biggins 2017), and local dynamics do not necessarily scale linearly up to broader regional patterns. Having data available from both focal studies and broad-scale investigations can help bridge these difficulties.

Pathogen monitoring in wildlife that is both large scale and long term is rare, yet the insights provided can be invaluable. The data reported here set the stage for future analyses that can examine *Y. pestis* associations with habitat type and potential shifts in distribution that have occurred over time because of climate or land use changes. *Y. pestis* associations with higher elevations are well documented (Eisen et al. 2007, 2008) and warming trends have already been detected at high elevations in the western United States (Rangwala and Miller 2012), signaling the potential for a change in higher elevation plague dynamics (Eads et al. 2020). Endemic *Y. pestis* is also often tied to semi-arid regions, similar to where it is believed to have evolved in the steppe regions of Asia (Spyrou et al. 2018). This is true in the United States as well, where plague is an introduced pathogen. First recorded in San Francisco in 1899, it spread relatively rapidly across the Western United States until it reached the area around the 100th meridian 40 years later, where its expansion came to a halt (Adjemian et al. 2007, Gage 2012). The 100th meridian is both where elevation begins to increase and where the shift from wet and humid East to dry and semi-arid West occurs in the contiguous United States. Recent work by Seager et al. (2018) examined how rising greenhouse gases may affect this arid-humid divide and their bias-corrected model finds that the shift from dry to wet now occurs further east, representing a recent expansion in aridity that may continue to move “progressively east as the century progresses.” The intersection between *Y. pestis* occurrence and climate are complex, so it remains to be seen if any shifts in temperature or hydroclimate would also be accompanied by a change in plague distribution to areas previously considered low risk for infection or plague free.

Enzootic *Y. pestis* can thrive in semi-arid regions, but conversely, anomalous precipitation events have been linked to pulses in primary productivity and subsequent increases in rodent hosts and flea vectors. Increases in rodent populations and their fleas can be tied to shifts from low-level transmission to explosive epizootic transmission or spillover into human populations, similar to patterns seen with Sin Nombre virus (Carver et al. 2015); however; there is also additional research that links epizootics to drought, where the lack of precipitation leaves rodent hosts in poor body condition, which may facilitate flea survival and plague transmission (Eads and Biggins 2017). These differential findings demonstrate that multiple pathways may lead to epizootics, but that the endpoint is the same, with epizootics representing an increased risk of spillover into people. The factors associated with spillover risk, however, may differ from the factors that drive enzootic transmission. Regardless, wildlife plague data are the only way to understand how this transmission determines where plague occurs on the landscape and to pinpoint risks before spillover events.

## Conclusion

Large, long-term datasets on *Y. pestis* exposure in wildlife, combined with recent advances in our understanding of plague dynamics and ecology, offer a path forward to better understand the ecology of plague and, in turn, the risks it poses to wildlife, domestic animal, and human health. This is applicable to many zoonotic pathogens, not just plague, and comprehensive baseline data like these can inform risk to human populations. As we have seen recently, outbreaks and pandemics require ecologists and modelers to produce results rapidly, often when the information and data needed to produce those results are severely limited (Getz et al. 2018). Proactive investment in surveillance and monitoring efforts can help produce those much-needed data in advance.
